# Lung ultrasound imaging can effectively monitor and guide treatment with pulmonary surfactant for preterm infants of pulmonary consolidation: a prospective observational cohort study

**DOI:** 10.3389/fped.2025.1634955

**Published:** 2025-10-14

**Authors:** Feng Zaili, Huang Na

**Affiliations:** ^1^ Department of Neonatology, The People's Hospital of Dehong Prefecture (The Affiliated Dehong Hospital of Kunming Medical University), Yunnan, China; ^2^ Graduate School, Pediatrics, The Kunming Medical University, Yunnan, China; ^3^ Department of Neonatology, The Dehong Vocational College, Yunnan, China

**Keywords:** preterm infants, neonatal respiratory distress syndrome, transient tachypnea of the newborn, lung ultrasound, pulmonary surfactant, pulmonary consolidation

## Abstract

**Background:**

A radiation-free method for dynamic evaluation of infant lung function is lung ultrasound (LUS).

**Objective:**

To assess if LUS-guided surfactant therapy, which is based on direct observation of pulmonary consolidation, is an effective way to guide treatment timing and dosage in preterm infants (less than 34 weeks of gestation).

**Methods:**

This prospective observational cohort study was conducted at Dehong Hospital affiliated to Kunming Medical University, in various clinical settings, including newborn wards, neonatal intensive care units (NICUs), obstetric operating rooms and delivery rooms. Within an hour of delivery, all preterm newborns received their first LUS. The frequency of monitoring was adjusted for those without consolidation at various intervals; if consolidation was detected, PS[Poractant alfa (Curosur®; Chiesi Farmaceutici S.p.A., Italy)] 200 mg/kg was administered immediately. A repeating dose of 100 mg/kg was administered if consolidation did not go away after 12 h. Following each PS treatment, LUS assessments were conducted every four hours, and monitoring was progressively decreased once consolidation was resolved. PS was not administered to TTN preterm newborns without consolidation. The frequency of PS administration, the prevalence of pulmonary consolidation, and the number of infants who had complete lung re-expansion were also noted.

**Results:**

With each PS dose, the rate of complete lung re-expansion among infants with neonatal respiratory distress syndrome (NRDS, *n* = 55) increased significantly (first dose: 60.0%, second dose: 63.6%, third dose: 75.0%; trend *χ*^2^ = 102.45, *P* < 0.001), with 96.4% of infants achieving complete re-expansion within three doses. On the other hand, regardless of dose escalation, children with transient tachypnea of the newborn (TTN, *n* = 37) achieved complete lung recruitment in 2 doses (*P* = 0.482), and all TTN infants eventually attained complete recruitment. The frequency of LUS examinations varied by clinical context, with the neonatal room having the lowest frequency (9.70%) and the obstetric operating room having the highest frequency (38.70%).

**Conclusion:**

In preterm newborns, NRDS and TTN can be effectively managed using LUS-guided PS therapy based on consolidation monitoring. While TTN can be treated with a fixed-dose regimen, NRDS has a dose-response relationship that supports customized dosage. The frequency and timing of surfactant administration in preterm newborns are determined by LUS-detected lung consolidation, which also acts as a decision-making indicator for PS therapy.

## Introduction

The hallmark pathophysiological signs of neonatal respiratory distress syndrome (NRDS), which is mostly caused by inadequate pulmonary surfactant (PS), include progressive alveolar collapse and atelectasis. The sole cure for the condition is early surfactant therapy, which also enhances results. lung ultrasound (LUS) has emerged as a key diagnostic technique for NRDS and for tracking lung recruitment in preterm infants (1, 2). For NRDS patients to have better clinical outcomes, early surfactant intervention is essential (3). On the other hand, high pulmonary fluid and poor lymphatic drainage are the hallmarks of transient tachypnea of the newborn (TTN), which results in alveolar fluid retention that usually doesn't call for surfactant treatment (4). Though it is still not widely used in clinical practice, new research indicates that surfactant therapy may have therapeutic promise for TTN-associated pulmonary consolidation (5–7).

The therapeutic utility of ultrasound-guided surfactant therapy has drawn more attention in recent years. Raschetti et al.'s quality improvement research (8) showed that ultrasound-guided therapy greatly increases the promptness of surfactant replenishment in premature newborns. The feasibility and safety of LUS-guided early surfactant replacement therapy in preterm infants with respiratory distress syndrome were validated by Rodriguez-Fanjul et al.'s randomized controlled trial (9). The diagnostic accuracy of LUS scoring systems in predicting surfactant requirements was thoroughly assessed by Capasso et al.'s (10) systematic review and meta-analysis, which also revealed ongoing discussions about the systems' standardization and efficacy.

The effectiveness and implementation techniques of this strategy are still up for debate, despite some research supporting the use of the LUS score to direct surfactant therapy (10–12). Standardized recommendations are anticipated to encourage uniformity in clinical practice, enable more prompt and accurate treatment, and ultimately enhance pediatric patient outcomes, according to the first international consensus guidelines for LUS-guided surfactant therapy (13). The shortcomings of the LUS scoring system, including its lack of consistent standardization, were specifically mentioned by Liu et al. (14). LUS scoring is still impacted by technical factors (such as probe frequency, imaging settings, operator experience) and clinical context (such as patient positioning, respiratory support mode) (14, 15), even with recent advancements in standardization and training, such as the development of international guidelines to improve practice consistency (2, 16) and the demonstration by Aichhorn et al. that standardized training improves inter-rater agreement (17). These drawbacks highlight the need to investigate more anatomically based and intuitive substitutes. In contrast to other research that mostly concentrated on LUS scores or particular ultrasound characteristics, this study presents a novel approach that uses dynamic monitoring and direct viewing of pulmonary consolidation to direct surfactant therapy. This strategy offers a real-time assessment method that is very compatible with the physiological objective of alveolar re-expansion.

To determine whether LUS-based direct viewing of pulmonary consolidation can successfully guide the timing and dosage of surfactant administration in preterm infants (less than 34 weeks of gestation), this prospective observational cohort study was conducted. We aim to make LUS a radiation-free, real-time tool for enhancing customized treatment plans by dynamically monitoring lung recruitment and reliably differentiating NRDS from TTN. The main goal is to assess if LUS-guided surfactant therapy, which is based on direct vision of pulmonary consolidation, can successfully direct treatment timing and dosage in preterm newborns (less than 34 weeks of gestation).

## Methods

### Study design

In accordance with the Statistical Analysis and Methods in Published Literature (SAMPL) Guidelines and the Strengthening the Reporting of Observational Studies in Epidemiology (STROBE) Statement, this study was carried out as a prospective observational cohort study. The Dehong People's Hospital Ethics Committee gave its approval to the study protocol (Approval No.: DYLL-KY2019002). Every procedure complies with the applicable institutional and national regulations.

### Sample size

The main objective of this prospective observational cohort study was to assess the viability and usefulness of LUS-guided surfactant therapy and monitoring in clinical settings. The study did not test pre-specified hypotheses between comparable groups; hence, statistical power-based formal sample size estimates were not carried out. All preterm children who met the inclusion criteria during the study period were included in the sample, as is customary for this type of exploratory methodological research.

### Study population

This study is a prospective observational study conducted at Dehong Hospital affiliated to Kunming Medical University. The study sites include the Level III newborn wards, neonatal intensive care units(NICUs), obstetric operating rooms and delivery rooms. The hospital's Medical Ethics Committee granted ethical approval (Approval No.: DYLL-KY2019002), and the guardians of the infants gave their informed consent. 217 preterm newborns (gestational age <34 weeks) born between March 2, 2019, and January 1, 2022, made up the group. 102 female and 115 male newborns with gestational ages between 26 and 34 weeks were part of the group. There were 120 cesarean sections and 97 vaginal deliveries. The weights of newborns ranged from 748 to 2,588 grams. Of the neonates involved, 55 experienced NRDS and 37 had transient total pulmonary hypertension (TTN).

### Inclusion and exclusion criteria

In this study, preterm infants (gestational age <34 weeks) are evaluated for PStherapy guided by LUS. According to age, gestational age, medical history, and status of informed consent, the criteria specify which infants are eligible and which are not.

#### Inclusion criteria

(1) Age at birth < 1 day; (2) Gestational age < 34 weeks; (3) Absence of fetal malformations or developmental abnormalities; (4) Consent for PStherapy; (5) No medical disputes between the hospital and guardians.

#### Exclusion criteria

(1) Death before lung recruitment, transfer to another hospital, or discontinuation of treatment; (2) Diaphragmatic hernia; (3) Lung sequestration; (4) Pneumothorax; (5) Infected or suspected infection: blood infection markers positive within 72 h of birth; positive blood or sputum cultures within 72 h; clinical or laboratory evidence of infection (e.g., maternal chorioamnionitis, positive blood culture, CRP >10 mg/L, leukocytosis/leukopenia); (6) Skin conditions impeding LUS examination; (7) Family history of PS allergy.

#### NICUs admission requirements

Was the site of this investigation. Participants were chosen based on their clinical requirement for a postpartum respiratory evaluation and their gestational age (less than 34 weeks). Institutional standards were followed for admission to NICUs: Every baby whose gestational age was less than 32 weeks was regularly moved to the NICUs for careful observation and preparation to offer breathing support. If an infant with a gestational age of ≥32–<34 weeks showed clinical indications of respiratory distress, needed supplemental oxygen (FiO_2_ > 21%), or required invasive or noninvasive breathing support, they were sent to the NICUs.

### Diagnostic criteria

Clinical presentation and LUS results are used to determine the diagnostic criteria for NRDS: TTN, and TTN with pulmonary consolidation (2, 4, 18, 19). The main clinical traits and LUS traits that set these three disorders apart are summarized in Table 1.

**Table 1:** Diagnostic criteria for NRDS and TTN diagnostic criteria for neonatal respiratory distress syndrome (NRDS), transient tachypnea of the newborn (TTN), and pulmonary consolidation secondary to TTN.

Criteria	NRDS	TTN	Pulmonary consolidation secondary to TTN
Clinical Presentation	Respiratory distress (tachypnea, grunting, retractions, nasal flaring) within 12 h of birth; May include cyanosis and three-concave signs.	Tachypnea (respiratory rate >60 breaths/min) within the first few hours of life; May include grunting, retractions, and mild cyanosis.	Clinical presentation consistent with TTN, but with the added finding of lung consolidation on LUS.
LUS Findings	Diffuse lung consolidation. Other findings may include pleural line abnormalities, absence of A-lines, heterogeneous echotexture, and pleural effusion.	Variable findings. May include: pulmonary interstitial syndrome, increased B-lines, heterogeneous echotexture, pleural effusion. Absence of lung consolidation.	Lung consolidation on LUS. Other findings may include pleural line abnormalities, absence of A-lines, heterogeneous echotexture, pleural effusion.

Diagnostic Criteria for Neonatal Respiratory Distress Syndrome (NRDS), Transient Tachypnea of the Newborn (TTN), and Pulmonary Consolidation Secondary to TTN Based on Clinical Presentation and Lung Ultrasound (LUS) Findings. The table outlines key clinical features and LUS characteristics to distinguish between these three conditions.

NRDS, neonatal respiratory distress syndrome; TTN, transient tachypnea of the newborn; LUS, lung ultrasound.

### LUS examination

A Mindray M9 portable color Doppler ultrasonography system (Shenzhen Mindray Biomedical Electronics Co., Ltd., China) fitted with a 9 MHz high-frequency linear array probe was used to perform LUS exams. Space compounding was turned off, and the scanning depth was adjusted to 4–6 cm with an emphasis on the pleura-lung interface. All LUS were performed by board-certified neonatologists (with LUS examination qualifications), who also prepare the diagnostic reports. Every region of both lungs should undergo longitudinal and transverse scans, with the probe always positioned perpendicular to the ribs or pleura (2, 16). The anterior and posterior axillary lines are typically used to split each lung into three sections: anterior, lateral, and posterior. This creates six bilateral zones for systematic scanning. Each lung is further separated into upper and lower lobes using the line that connects the two nipples if the patient weighs more than 3 kg. This results in twelve areas on each side. In supine, lateral, and prone postures, a thorough evaluation of the anterior, lateral, and posterior regions should be part of the scanning process.

Newborns are quickly stabilized and resuscitated after birth, and then they are promptly evaluated for LUS in the newborn wards, neonatal intensive care units (NICUs), obstetric operating rooms and delivery rooms. If there is any pulmonary consolidation on LUS, regardless of size or quantity, PS therapy is started right away as long as it is verified in two or more imaging planes and continues even after positional adjustments.

### Guidelines for the administration of PS via LUS

**Figure 1:**
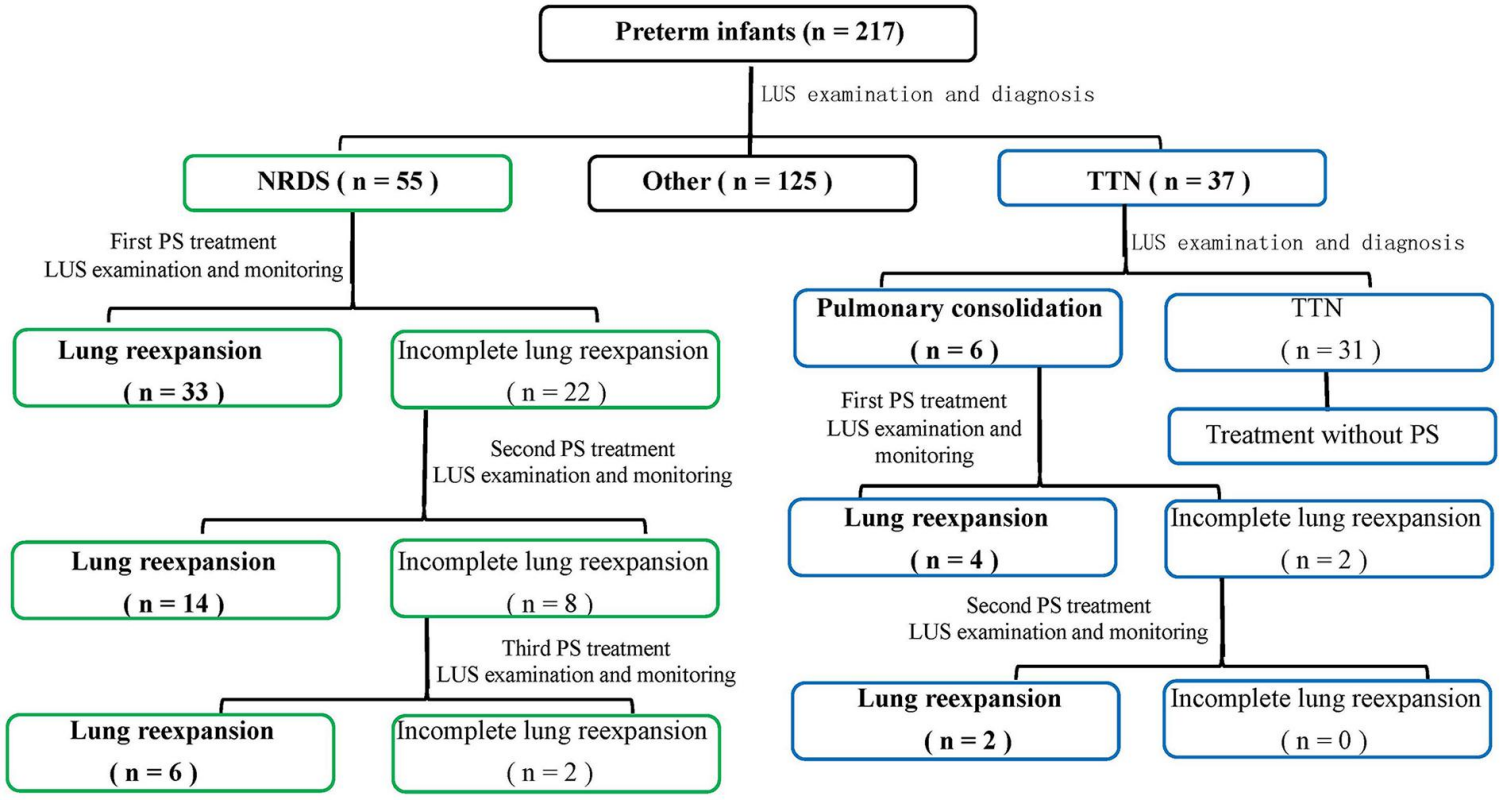
Algorithm for LUS-guided surfactant therapy in preterm infants. This flowchart outlines the decision-making process for surfactant administration based on serial lung ultrasound (LUS) assessments. It includes initial LUS evaluation, criteria for surfactant initiation (e.g., lung consolidation), dosing, monitoring frequency, retreatment criteria, and ventilator management strategies.

Within an hour of birth, all preterm newborns had their initial LUS assessment, as indicated in Figure 1 and Table 2. LUS monitoring was carried out every 4 h until 24 h postnatal age, after which it was modified to every 8 h if no lung consolidation was found. After 72 h of life, monitoring was switched to once daily for babies with TTN who did not have lung consolidation. Poractant alfa (Curosur®; Chiesi Farmaceutici S.p.A., Italy) was started right away at a loading dose of 200 mg/kg if LUS showed pulmonary consolidation, regardless of the diagnosis [TNN with consolidation or neonatal respiratory distress syndrome (NRDS)]. Additional doses of 100 mg/kg could be given every 12 h if consolidation continued. LUS assessments must be conducted every four hours after each PS dose until consolidation is fully resolved. The frequency of post-resolution monitoring should be tapered to occur every 8 h on Day 1, every 12 h on Day 2, and then every day after that. PS therapy and the previously described specialist post-treatment monitoring are not necessary for infants with TTN who do not have consolidation. At every LUS examination, respiratory support modes—such as invasive mechanical ventilation (volume-guaranteed mode), noninvasive ventilation (CPAP), and high-flow nasal cannula—were recorded and provided in accordance with clinical indications. For newborns receiving CPAP support who are breathing on their own, PSis mostly given by “minimally invasive surfactant administration” (LISA). Porcine lung phospholipids (Curosurf®) are gradually infused into the trachea using a 5-Fr feeding tube that is guided by a laryngoscope. PS is given through the endotracheal tube during mechanical ventilation for newborns who need to be intubated because of apnea or severe respiratory failure. The full resolution of all lung consolidation on LUS, with the return of normal A-lines and smooth, regular pleural lines in all scanned lung fields, was referred to as complete alveolar re-expansion. A decrease in the quantity or magnitude of consolidations without full resolution was referred to as partial re-expansion.

**Table 2:** LUS-guided pulmonary surfactant (PS) administration protocol in preterm infants.

Condition	NRDS	TTN with pulmonary consolidation	TTN without pulmonary consolidation
Initial LUS Examination	Within the first hour after birth	Within the first hour after birth	Within the first hour after birth
LUS Frequency without Consolidation (after birth)	Every 4 h until 24 h of age; then every 8 h	Every 4 h until 24 h of age; then every 8 h	Every 4 h until 24 h of age; then every 8 h until 72 h of age; then once daily thereafter
LUS Frequency after PS Treatment (with consolidation)	Every 4 h after each PS dose until consolidation resolves	Every 4 h after each PS dose until consolidation resolves	Not applicable
LUS Frequency after Consolidation Resolves	Every 8 h on the first day; every 12 h on the second day; once daily from the third day onward	Every 8 h on the first day; every 12 h on the second day; once daily from the third day onward	Not applicable
Pulmonary Surfactant (PS) Dosage and Administration	Initial dose: 200 mg/kg; subsequent doses: 100 mg/kg, at least 12 h apart	Initial dose: 200 mg/kg; subsequent doses: 100 mg/kg, at least 12 h apart	Not applicable

LUS-Guided Pulmonary Surfactant (PS) Administration Protocol in Preterm Infant. Protocol for guiding surfactant therapy using lung ultrasound (LUS) in preterm infants with NRDS or TTN with pulmonary consolidation. Initial LUS within first hour after birth. Monitoring frequency adjusted by consolidation and postnatal age. Surfactant (e.g., Poractant alfa) dosing: initial 200 mg/kg, then 100 mg/kg at ≥12 h intervals.

NRDS, neonatal respiratory distress syndrome; TTN, transient tachypnea of the newborn; LUS, lung ultrasound; PS, pulmonary surfactant.

### Statistical analysis

The findings of the Kruskal–Wallis *H* test, which examines variations in the distribution of gestational ages among initial LUS tests in four clinical contexts (the newborn wards, NICUs, obstetric operating rooms and delivery rooms), are shown in Table 3. Pairwise comparisons were conducted using the Mann–Whitney *U* test with Bonferroni adjustment, where statistically significant differences were found. The linear relationship between the diagnosis of LUS and gestational age in preterm newborns was examined using the Cochran-Armitage trend test, as indicated in Table 4.

**Table 3:** Distribution of initial LUS examinations by clinical setting and gestational age (*N* = 217) [*n* (%)].

Clinical setting	Gestational age group (weeks)				
	<28	≥28–<30	≥30–<32	≥32	Total
Obstetric Delivery Room	8 (3.69)	14 (6.45)	18 (8.29)	32 (14.75)	72 (33.20)
Obstetric Operating Room	10 (4.61)	16 (7.37)	22 (10.14)	36 (16.59)	84 (38.70)
Neonatal Room	0	0	3 (1.38)	18 (8.29)	21 (9.70)
NICU	3 (1.38)	6 (2.76)	9 (4.15)	22 (10.14)	40 (18.40)
*H* = 15.82					
*P* = 0.0012					

Kruskal–Wallis *H* test analysis.

Distribution of Initial Lung Ultrasound (LUS) Examinations by Clinical Setting and Gestational Age (*N* = 217).

This table illustrates the distribution of initial LUS examinations across different clinical settings (Obstetric Delivery Room, Obstetric Operating Room, Neonatal Room, NICU) stratified by gestational age groups (<28, ≥28–<30, ≥30–<32, ≥32 weeks).

NICU, neonatal intensive care unit; LUS, lung ultrasound.

**Table 4:** Analysis of the correlation between LUS diagnosis and gestational age distribution in preterm infants (*N* = 217).

Diagnosis	<28 weeks (%)	≥28–<30 weeks (%)	≥30–<32 weeks (%)	≥32–<34 weeks (%)	*χ*^2^ value	*p*-value (Trend)
NRDS	21 (9.68)	19 (8.76)	11 (5.07)	4 (1.84)	115.31	<0.001
TTN	0 (0.00)	4 (1.84)	9 (4.15)	24 (11.06)	18.32	<0.001
Normal LUS	0 (0.00)	12 (5.53)	27 (12.44)	73 (33.64)	102.45	<0.001
Other Diagnoses	0 (0.00)	1 (0.46)	5 (2.30)	7 (3.22)	2.34	0.123

Association Between LUS Diagnoses and Gestational Age Distribution in Preterm Infants (*N* = 217).

This table analyzes the correlation between LUS diagnoses (NRDS, TTN, Normal LUS, Other Diagnoses) and gestational age categories (<28, ≥28–<30, ≥30–<32, ≥32–<34 weeks) in preterm infants. Percentages reflect the proportion of infants within each gestational age group diagnosed with each condition. Chi-square tests evaluated trends across gestational age categories, with *p*-values indicating statistical significance (*p* *<* *0.05*). Missing data (e.g., NRDS cases in ≥32–<34 weeks) are denoted as 0%.

NRDS, neonatal respiratory distress syndrome; TTN, transient tachypnea of the newborn; LUS, lung ultrasound.

The relationship between surfactant dose requirements and complete lung recruitment rates in infants with NRDS: and TTN was examined using the Cochran-Armitage trend test for Table 5, determining whether recruitment rates significantly improved with increasing dose. The relationship between surfactant dose requirements and full lung recruitment in TTN newborns was examined using Fisher's exact test, as shown in Table 6. To determine the significance of efficacy differences between dose groups in the limited sample (*n* = 6), exact binomial distribution probabilities were computed. A two-tailed significance threshold of 0.05 was used for all tests.

**Table 5:** Surfactant dose requirements for complete lung re-expansion in NRDS infants (*n* = 55).

Dose number	Complete re-expansion (*n*, %)	Incomplete re-expansion (*n*, %)	Total (*n*)	*χ*^2^ trend	*p*-value
1st	33 (60.00)	22 (40.00)	55	102.45	0.0000
2st	14 (63.64)	8 (36.36)	22		
3st	6 (75.00)	2 (25.00)	8		
Overall	53 (96.36)	2 (3.64)	55		

Surfactant Dosage and Complete Lung Re-expansion in NRDS Infants (*n* = 55).

This study examined the association between surfactant dose (first, second, and third dose) and complete lung recruitment in infants with NRDS. The chi-square trend test (*χ*^2^ = 102.45, *p* < 0.001) indicated a significant dose-response relationship between surfactant dose and treatment efficacy.

NRDS, neonatal respiratory distress syndrome.

**Table 6:** Association between surfactant treatment response and dosage in infants with TTN.

Dose number	Complete re-expansion (*n*, %)	Incomplete re-expansion (*n*, %)	*p*-value (Fisher's exact test)
1st	4 (66.67)	2 (33.33)	0.482
2st	2 (100.00)	0 (0.00)	
Overall	6 (100.00)	0 (0.00)	

Surfactant Treatment Response vs. Dosage in TTN Infants (*n* = 6).

This table evaluates the impact of surfactant dosage (1st, 2nd) on treatment response in infants with TTN. Fisher's Exact Test (*p* = 0.482) showed no statistically significant association between dose and outcome.

TTN, transient tachypnea of the newborn.

## Results

The distribution of gestational ages at initial LUS examinations varied significantly across the four clinical settings, as indicated in Table 3 (*H* = 15.82, *d* = 3, *p* < 0.001). Additional paired comparisons showed.

Comparisons between the remaining groups revealed no statistical significance, although there were significant differences between the Obstetric Delivery Room and the Neonatal Room (*p* < 0.006) and between the Obstetric Operating Room and the Neonatal Room (*p* < 0.006) (Table 5). These results show that the clinical context after birth affects the gestational age distribution of neonates needing an initial LUS examination. Professionals with the skills to conduct LUS tests and the required tools should always be on hand in these high-risk locations.

**Figure 2:**
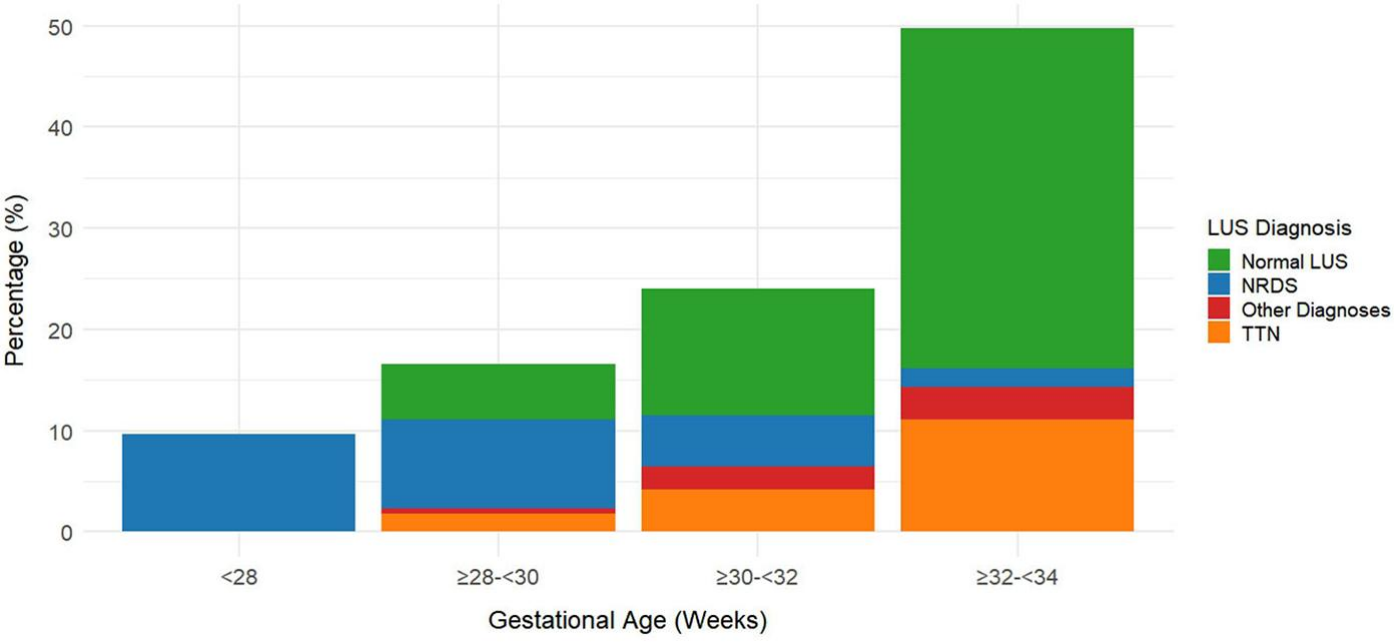
Distribution of lung ultrasound (LUS) diagnoses by gestational age in preterm infants (*N* = 217). This figure shows the distribution of lung ultrasound (LUS) diagnoses across gestational age groups in preterm infants. The proportion of neonatal respiratory distress syndrome (NRDS) decreases with increasing gestational age, while the proportions of normal LUS findings and transient tachypnea of the newborn (TTN) increase.

Table 4 presents the association analysis between gestational age and LUS diagnosis, which is visualized in Figure 2. Neonatal respiratory distress syndrome (NRDS) frequency among preterm newborns with gestational age <34 weeks significantly decreased with increasing gestational age [*χ*^2^_(trend) = 24.67, *p* < 0.001], as seen in Table 4 and the stacked percentage bar chart (Figure 2). As gestational age grew, the incidence of TTN increased considerably [*χ*^2^_(trend) = 18.32, *p* < 0.001]. With gestational age, the incidence of normal lung ultrasound (LUS) rose significantly [*χ*^2^_(trend) = 102.45, *p* < 0.001]. The basis for individualized diagnosis and treatment approaches is the reliance of TTN and NRDS on gestational age.

**Figure 3:**
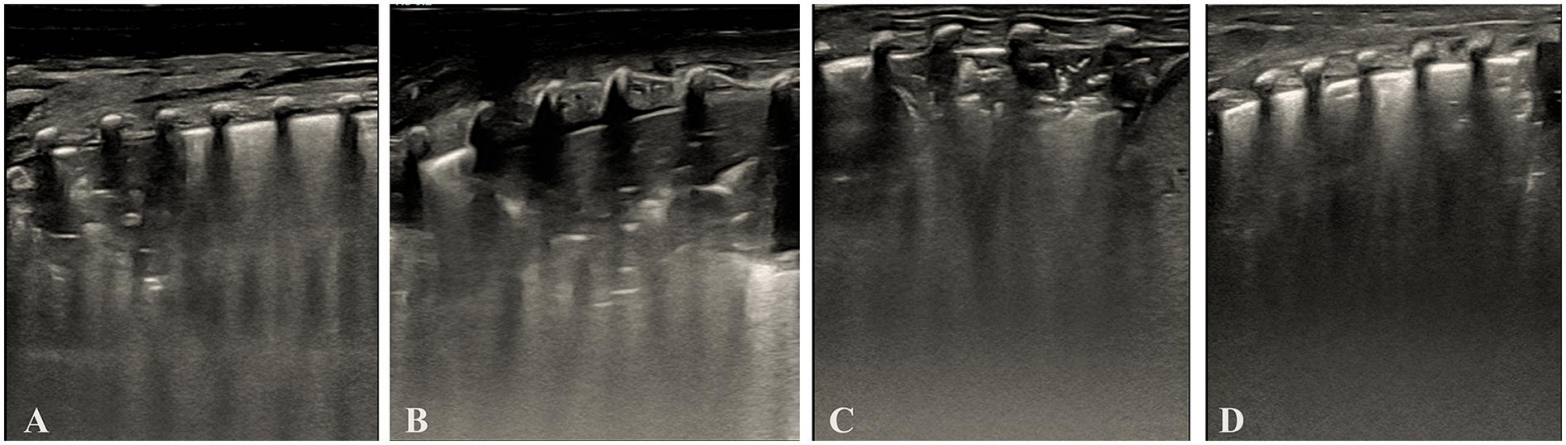
Illustrates sequential LUS findings in an NRDS infant. **(Panel A)** Initial LUS showing pulmonary consolidation, bronchial inflation sign, absent A-lines, and irregular pleural line. **(Panel B)** LUS after the first dose of surfactant, showed persistent consolidation, bronchial inflation sign, absent A-lines, and irregular pleural line. **(Panel C)** LUS after the second dose of surfactant, showed reduced consolidation, bronchial inflation sign, absent A-lines, and irregular pleural line. **(Panel D)** LUS after the third dose of surfactant showed complete resolution of consolidation with a normal pleural line and visible A-lines. Images were acquired using a Mindray M9 portable color Doppler ultrasound system (Shenzhen Mindray Biomedical Electronics Co., Ltd., China).

As shown in Table 5. Dose-effect trend: Complete re-expansion rates increased significantly with escalating doses [*χ*^2^_(trend) = 102.45, *p* < 0.001]. 1st dose: 60.00% (33/55), 2nd dose: 63.64% (14/22), 3rd dose: 75.00% (6/8). Overall success rate: 96.36% (53/55) of infants achieved complete lung re-expansion after ≤3 doses. The dose of surfactant showed a significant positive correlation with the complete re-expansion rate in NRDS infants, providing evidence-based support for individualized dosing regimens. Figure 3 illustrates sequential LUS findings in an NRDS infant.

**Figure 4:**
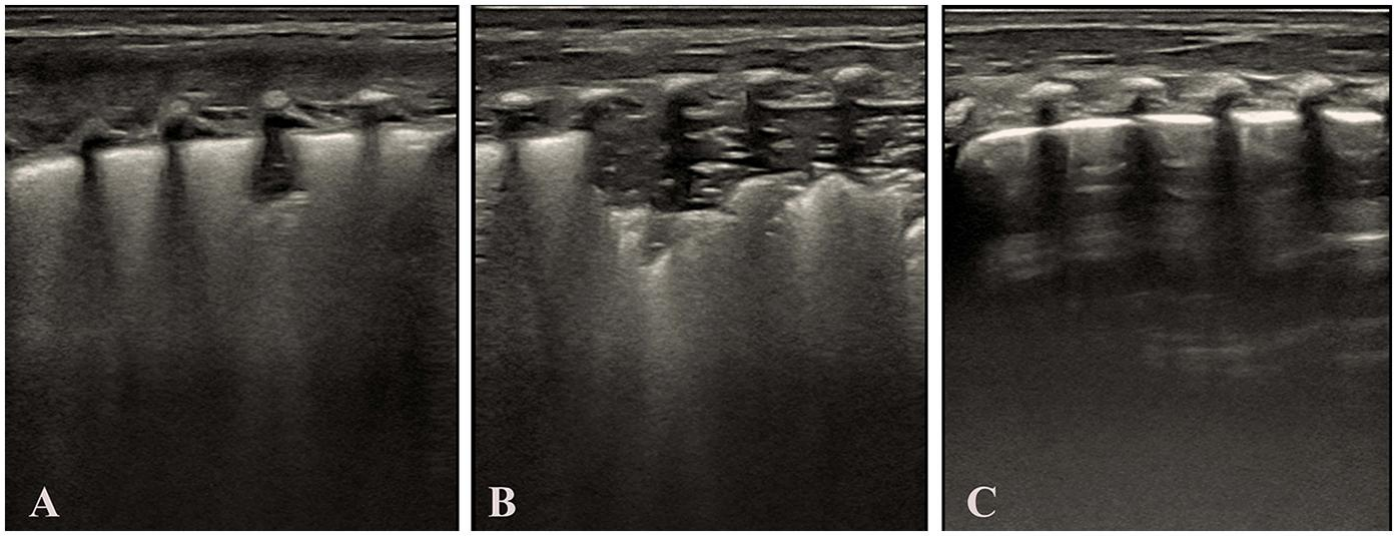
Demonstrates LUS imaging and PS treatment response of TTN infant with pulmonary consolidation. **(Panel A)** Initial LUS demonstrates features consistent with the alveolar interstitial syndrome (AIS), including a blurred pleural line and absence of consolidation. **(Panel B)** Subsequent LUS shows the development of pulmonary consolidation, bronchial inflation sign, absent A-lines, and an irregular pleural line. **(Panel C)** LUS following surfactant administration, showing resolution of consolidation, a normal pleural line, and the reappearance of A-lines. Images were acquired using a Mindray M9 portable color Doppler ultrasound system (Shenzhen Mindray Biomedical Electronics Co., Ltd., China).

As shown in Table 6. Dose-effect Analysis: 1st Dose Group: Complete re-expansion rate of 66.67% (4/6). 2nd Dose Group: Complete re-expansion rate of 100.00% (2/2). Fisher's exact test demonstrated no significant association between dose escalation and complete re-expansion (*p* = 0.482). Surfactant treatment is safe and effective for TTN infants, but dose escalation does not significantly improve outcomes. Despite the small sample size, all infants achieved complete lung re-expansion within ≤2 doses, suggesting surfactant therapy is effective for TTN-associated pulmonary consolidation. Larger studies are needed to validate dose-dependent effects. Figure 4 demonstrates LUS imaging and PS treatment response of TTN infant with pulmonary consolidation.

## Discussion

According to our prospective observational cohort study, newborn wards, NICUs, obstetric operating rooms and delivery rooms can all benefit from the use of lung ultrasound (LUS). It can help diagnose NRDS: TTN, and other pulmonary abnormalities in preterm newborns. It is suitable for evaluating and tracking preterm infants at various gestational ages. For the monitoring and diagnosis of infant pulmonary disorders, lung ultrasound (LUS) is more portable, radiation-free, non-invasive, and easily accessible at the bedside than chest radiography (20). While chest radiography only offers static lung pictures at a certain moment in time, LUS's real-time imaging capacity allows for dynamic monitoring of pulmonary ventilation and surfactant therapy response.

In line with the well-established pathophysiological mechanism of surfactant deficiency in immature lungs, our prospective observational cohort study shows a significant inverse correlation between gestational age and the incidence of NRDS. On the other hand, TTN is more common in late preterm and term infants, and its occurrence positively corresponds with gestational age. Additionally, as gestational age grows, the percentage of babies with normal LUS findings rises as well, together indicating improved pulmonary maturity—a typical phase of fetal development. Overall, our results highlight the potential use of LUS in differentiating between non-pulmonary diseases and prevalent respiratory disorders (e.g., TTN and NRDS) across various gestational age groups.

Lung ultrasound (LUS) can direct surfactant therapy in preterm infants with TTN-associated consolidation and newborn respiratory distress syndrome (NRDS), as our prospective observational cohort study shows. The results show that managing surfactant therapy in preterm infants with respiratory distress can be accomplished with a LUS-guided approach based on direct observation of pulmonary consolidation. With increasing PS dosages, we saw a marked rise in the frequencies of full lung re-expansion in NRDS infants, confirming the usefulness of LUS in tracking therapy response and directing dose escalation. Infants with TTN-associated consolidation, on the other hand, showed full re-expansion in a small number of doses, indicating that fixed-dose regimens may be useful in this population.

Numerous studies have shown that the LUS score system, a semi-quantitative method, may accurately predict surfactant requirements (10–12, 21). Inter-observer variability and sensitivity to technical settings are two drawbacks of its use, though (14, 15). Technical elements, including probe frequency, imaging conditions, and operator experience, may have an impact on the validity and reproducibility of LUS scoring (14, 15). High-frequency probes, for example, improve near-field resolution but restrict deep-field penetration, which may have an impact on B-line quantification.

Furthermore, LUS performance and score reliability may be affected by clinical circumstances, such as respiratory assistance, patient posture, and underlying disease (15). Although there are still issues with utilizing LUS scoringto direct PS therapy for preterm newborns, standardization and validation have advanced significantly in recent years. For example, research by Aichhorn et al. (17) showed that after standardized training, inter-rater reliability improved. Additionally, international guidelines (2, 16) have been developed to encourage uniformity in practice.

Our study proposes and validates a technique based on direct anatomical visualization (consolidation). Some of the acknowledged drawbacks of the LUS scoring system are addressed in our study: Dynamic changes in consolidation can be directly related to treatment response, which facilitates bedside decision-making. Direct observation of consolidation resolution gives clinicians immediate physiological input, potentially minimizing subjective interpretation. Compared to research that uses LUS scoringmethods, our results are different. Our methodology offers direct anatomical feedback on pathological (consolidation) resolution, whereas scoring systems provide semi-quantitative evaluations. This directly corresponds to the physiological objective of alveolar re-expansion and might be more intuitive for doctors.

Therefore, our study indirectly adds to the current debate about the need for additional standardization and validation of LUS scoring systems by providing an alternate guidance technique. To elucidate their various roles in clinical practice, we urge future research to compare these approaches head-to-head. It is crucial to remember that the purpose of this study was not to directly compare established LUS scoringsystems with consolidation-based recommendations. As a result, we do not support the idea that one approach is better than another. For clinical decision-making, however, concentrating only on pulmonary consolidation might provide a more logical, anatomy-based option. Consolidation-based and score-based techniques must be explicitly compared in future multicenter randomized trials to ascertain their respective benefits.

### Innovative features

Novel Features of This Research. Our study shows that surfactant administration in NRDS should be carried out under LUS guidance and sustained until alveolar re-expansion is confirmed based on the presence of pulmonary consolidation. Subsequent LUS monitoring indicates that when LUS identifies secondary pulmonary consolidation in TTN, surfactant therapy should be started. According to this study, LUS demonstrates remarkable adaptability and efficacy in a variety of therapeutic contexts.

### Limitations

There are a number of constraints to be aware of: First, the results may not be as broadly applicable as they may be because this is a single-center study. Second, conclusive causal inferences are not possible due to the observational design. Third, the strength of subgroup analysis was constrained by the TTN group's comparatively small sample size (*n* = 37). Fourth, rather than following established procedures, the kind and level of respiratory assistance employed in this trial were customized for each patient based on their clinical needs. This could have an impact on lung ventilation and ultrasound signs of lung consolidation, which could be confounding variables.

## Conclusion

All things considered, direct observation of lung consolidation using LUS provides a useful and efficient way to direct the amount and timing of surfactant therapy in preterm newborns. Through dose-response monitoring, this method allows for customized management of NRDS and could streamline TTN support procedures. To directly compare our consolidation-based approach with well-established LUS scoringmethods, multicenter randomized trials are required in the future.

## Data Availability

The datasets presented in this article are not readily available because No restrictions. Requests to access the datasets should be directed to No dataset.
